# Oxidative Stress and Lipid Peroxidation Products in Cancer Progression and Therapy

**DOI:** 10.5402/2012/137289

**Published:** 2012-10-17

**Authors:** Giuseppina Barrera

**Affiliations:** Department of Medicine and Experimental Oncology, University of Turin, Corso Raffaello 30, 10125 Torino, Italy

## Abstract

The generation of reactive oxygen species (ROS) and an altered redox status are common biochemical aspects in cancer cells. ROS can react with the polyunsaturated fatty acids of lipid membranes and induce lipid peroxidation. The end products of lipid peroxidation, 4-hydroxynonenal (HNE), have been considered to be a second messenger of oxidative stress. Beyond ROS involvement in carcinogenesis, increased ROS level can inhibit tumor cell growth. Indeed, in tumors in advanced stages, a further increase of oxidative stress, such as that occurs when using several anticancer drugs and radiation therapy, can overcome the antioxidant defenses of cancer cells and drive them to apoptosis. High concentrations of HNE can also induce apoptosis in cancer cells. However, some cells escape the apoptosis induced by chemical or radiation therapy through the adaptation to intrinsic oxidative stress which confers drug resistance. This paper is focused on recent advances in the studies of the relation between oxidative stress, lipid peroxidation products, and cancer progression with particular attention to the pro-oxidant anticancer agents and the drug-resistant mechanisms, which could be modulated to obtain a better response to cancer therapy.

## 1. Oxidative Stress and Lipid Peroxidation

A body of evidence suggests that oxidative stress and resulting lipid peroxidation are involved in various and numerous pathological states including inflammation, atherosclerosis, neurodegenerative diseases, and cancer. The term “oxidative stress” is frequently used to describe the imbalances in redox couples such as those reduced to oxidized glutathione (GSH/GSSG) or NADPH/NADP^+^ ratios. Such metabolic disturbances not only involve the overproduction of reactive free radicals but also occur via a nonfree radical pathway, for example, by hydrogen peroxide [1]. In such cases, the products of its action are molecules that are enriched in one or more oxygen atoms that are generally considered to be markers of oxidative stress [2].

Reactive oxygen species (ROS) are thought to be the major ones responsible for the alteration of macromolecules which is often termed oxidative stress. ROS are generated as by-products of cellular metabolism, primarily in the mitochondria [3] and include free radicals such as superoxide anion (O_2_^−•^), perhydroxyl radical (HO_2_^•^), hydroxyl radical (^•^OH), nitric oxide (NO), and other species such as hydrogen peroxide (H_2_O_2_), singlet oxygen (^1^O_2_), hypochlorous acid (HOCl), and peroxynitrite (ONOO^−^) [4]. The hydroxyl radical ^•^OH is the most reactive radical that can arise through the Fenton reaction and the Haber-Weiss reaction from hydrogen peroxide and metal species (iron, copper) [5, 6].

To prevent the damage from ROS, cells possess several antioxidant enzymes such as superoxide dismutases MnSOD and Cu/ZnSOD, which are located in the mitochondria and the cytosol, respectively, where they convert superoxide into hydrogen peroxide [7]. The decomposition of hydrogen peroxide to water and oxygen is further catalyzed by catalase. Another antioxidant defense mechanism includes nonenzymatic antioxidants such as glutathione (GSH), which functions in the cellular thiol/disulfide system [8, 9].

The reactive intermediates, produced by oxidative stress, can alter the membrane bylayers and cause the lipid peroxidation of polyunsaturated fatty acids (PUFA) leading to the formation of lipoperoxyl radical (LOO^•^), which, in turn, reacts with a lipid to yield a lipid radical and a lipid hydroperoxide (LOOH). LOOHs are unstable: they generate new peroxyl and alkoxy radicals and decompose to secondary products [10–12]. Such free radicals produced during lipid peroxidation have some very local effects, because of their short life, but the breakdown products of lipid peroxides may serve as “oxidative stress second messengers,” due to their prolonged half-life and their ability to diffuse from their site of formation, compared to free radicals. Those breakdown products, mostly aldehydes, such as malonaldehyde, hexanal, 4-hydroxynonenal, or acrolein have received a lot of attention because they are the most reactive compounds [13].

The lipid peroxidation and the breakage of lipids with the formation of reactive compounds can lead to changes in the permeability and fluidity of the membrane lipid bilayer and can dramatically alter cell integrity [5].

Among the products of lipid peroxidation, 4-hydroxynonenal (HNE), is the most intensively studied [13, 14] since it is a highly electrophilic molecule that easily reacts with low-molecular-weight compounds, such as glutathione, with proteins and, at higher concentration, with DNA [15, 16]. Due to its chemical reactivity, this breakdown product can make covalent modifications on macromolecules and exert some biological effects. The reactivity of HNE depends on three main functional groups: the aldehyde group, the C=C double bond, and the hydroxyl group, which can participate, alone or in sequence, in chemical reactions with other molecules [15]. It has been demonstrated that HNE modifies proteins, either by forming simple Michael adducts with lysyl, histidyl, and cysteinyl residues [17] or through Schiff base formation with lysyl residues, leading to pyrrole formation [18]. In addition, HNE modification can result in cross-linking of two lysyl residues through reversibly formed Schiff base Michael adducts [19]. Inside the cells, the amount of HNE, and of consequence of HNE protein adducts, represents a steady-state concentration between the aldehyde produced and that is catabolized. Indeed, once formed, HNE is rapidly degraded by three major reactions: reduction to 1,4-dihydroxy-2-nonene by alcohol dehydrogenases, oxidation to 4-hydroxy-2-nonenoic acid by aldehyde dehydrogenase, or formation of the glutathione conjugate (GS-HNE) catalyzed by glutathione S-transferases. The majority of HNE is metabolized through forming GS-HNE [20]. 

## 2. Levels of Oxidative Stress and Lipid Peroxidation Products in Cancer Cells

In recent years, it has become evident that compared with their normal counterparts, many types of cancer cells have increased levels of ROS [21, 22]. For example, leukaemia cells freshly isolated from blood samples from patients with chronic lymphocytic leukaemia or hairy-cell leukaemia showed increased ROS production compared with normal lymphocytes [23, 24]. In solid tumours, studies have shown increased levels of oxidative damage products, such as oxidized DNA base (8OHdG), which is the most frequently investigated product, because of its mutagenic character and the high sensitivity of its immunological detection [25]. The increase in 8OHdG has been demonstrated in thyroid neoplasia [26], in squamous cell carcinoma [27], in non-small-cell lung cancer [28], and in prostate cancer cells [29].

A moderate increase in ROS can promote cell proliferation and differentiation [30, 31], whereas excessive amounts of ROS can cause oxidative damage. Therefore, maintaining ROS homeostasis is crucial for normal cell growth and survival. An increase in ROS is associated with abnormal cancer cell growth and reflects a disruption of redox homeostasis due either to an elevation of ROS production or to a decline of ROS-scavenging capacity [32]. Indeed, the levels of ROS-scavenging enzymes such as SOD, glutathione peroxidase, and peroxiredoxin have been shown to be significantly altered in malignant cells [33] and in primary cancer tissues [34–36], suggesting aberrant regulation of redox homeostasis and stress adaptation in cancer cells. The increase of ROS production may depend on diverse mechanisms, such as the activation of oncogenes, aberrant metabolism, mitochondrial dysfunction, and loss of functional p53 [37–39]. Growth factors and cytokines too stimulate the production of ROS to exert their diverse biological effects in cancer [40, 41]. Moreover, many cancers arise from sites of chronic irritation, infection, or inflammation, which is a critical component of tumor progression. Production of ROS by inflammatory cells as neutrophils and macrophages is well established, and it represents a mechanism to kill tumor cells. For example, tumour-associated macrophages were shown to induce sublethal oxidative stress in murine mammary tumour cells, possibly through the secretion of the inflammatory cytokine tumour necrosis factor-*α* (TNF-*α*) [42]. In neutrophils and macrophages, a rapid burst of superoxide formation primarily mediated by NAPDH oxidase leads to subsequent production of hydrogen peroxide which, in turn, can lead to oxidative stress-induced cell death [43].

Although an increase of oxidative stress has been demonstrated in the majority of cancer types, the concentration of lipid peroxidation products in cancer cells is yet a matter of debate. First experiments in this field demonstrated that in the hepatoma cells the level of lipid peroxidation products was lower than in normal liver cells [44] and depended on the degree of deviation. According to these results, Canuto et al. [45] demonstrated that during rat liver carcinogenesis, the activities of the enzymes metabolizing the aldehydes increased, thus rendering the cancer cells more protected against the cytotoxic effect of aldehydes. Moreover, in hepatoma cells, the major part of HNE is converted to HNE-GSH conjugate which is rapidly and efficiently exported out of the cell [46].

The analysis of HNE-protein adducts in different types of kidney tumors demonstrated that these adducts occur in kidneys in both normal and tumor cells, although immunomorphologic analyses suggest less HNE-protein adducts in tumor cells [47]. *In vivo* studies on human colon adenocarcinoma at different TNM and G staging showed a decrease of HNE in cancer colon biopsies with respect to normal surrounding tissues [48]. To the contrary, other experimental results demonstrated that the lipid peroxidation products, malondialdehyde and HNE, were increased in colorectal cancer tissues [49].

In thyroid tumors, with a high level of oxidative stress, both the HNE and 8OHdG content (as a marker of oxidative stress) was significantly higher than in matched normal tissue [50]. Analogously, lipid peroxidation seems to be a common pathological process in astrocytic and ependymal glial tumors, in which the incidence of HNE-immunopositive tumor cells increased with increasing grades of malignancy [51]. In other tumor types such as the breast cancers at different degrees of malignancy, 8-OHdG expression diminished significantly in invasive breast carcinomas compared to noninvasive lesions; conversely, HNE immunostaining was strongest in invasive breast carcinomas [52].

These divergent results about the concentration of HNE in tumors of different origins and the discrepancy between the level of oxidative stress and the level of products of lipid peroxidation could have diverse causes: the pattern of HNE-metabolizing enzymes in the tumor cells, the lipid composition of the cell membranes with a different level of peroxidizable substrates, such as PUFA, and the presence of inflammatory cells, which can increase the level of diffusible HNE from the tumor surrounding tissues.

However, although the concentration of HNE in the tumor cells not always correlated with the level of oxidative stress, in the majority of cancer cells, HNE is a common denominator in the enhancement of oxidative stress caused by H_2_O_2_, superoxide, UV, heat, and oxidant chemicals such as doxorubicin [53].

## 3. Biological Effects of ROS in Cancer Cells

ROS can elicit a broad spectrum of responses depending on the magnitude of the level, the duration of exposure, the localization, and the nature of ROS involved [54]. In general, low levels of ROS are mitogenic and promote cell proliferation and survival, while intermediate levels cause transient or permanent cell cycle arrest and induce cell differentiation [55]. At high levels, ROS can easily react with membrane lipids, causing an alteration of membrane permeability; with DNA, causing damage and genomic instability; with proteins, causing oxidative modifications which might result in catalytically less active enzymes or proteins more susceptible to proteolytic degradation. In this case ROS are detrimental and induce cell apoptosis or necrosis [56, 57]. On the other hand, when ROS production does not irreversibly alter cell viability, they can act as a primary messenger, modulating several intracellular signalling cascades leading to cancer progression. Indeed, it has been demonstrated that ROS activate the pathways of mitogen-activated protein kinases (MAPKs), phosphatidylinositol-3-kinase (PI3K]/Akt), phospholipase C-g1 (PLCg1), protein kinase C, nuclear factor-*κ*B (NF-*κ*B), and Jak/Stat [55–59]. Moreover, through distinct signal transduction cascades, ROS can induce the expression of families of heat shock proteins, immediate early genes of the bZIP family members like c-Jun and c-Fos, hypoxia-inducible factor (HIF), and antioxidative enzymes which help to regulate redox homeostasis, the expression of transforming oncoproteins, and growth factors [54]. ROS have been involved in the control of cell cycle progression also. Regulation of the cell cycle requires the precise integration of many different processes in cells, including the signal transduction cascades activated by mitogens and the extracellular matrix, the ubiquitination processes and subsequent degradation of proteins in the proteasome, and the proper (re-)organization of the cytoskeleton, in particular actin and tubulin, amongst many others [60]. Since it has been demonstrated that ROS influence many of these processes, it is obvious that ROS influence cell cycle progression [60]. However, in these studies, most of evidence indicates that the ROS action leads to a blocking of cell cycle progression. Several studies have demonstrated that hyperoxia induces inhibition of proliferation in G1, S, and G2 phases of the cell cycle [61–63]. A decrease in cell cycle progression due to H_2_O_2_ was also observed in Chinese hamster ovary cells when H_2_O_2_ was added to the cells during the M phase [64]. Hyperoxia (95% O_2_, 5% CO_2_) caused an increase in p53 expression and phosphorylation, p21 mRNA and protein expression and cell cycle arrest in HCT116 colon carcinoma cells. In contrast, no effects on p21 expression were observed in either p53- or p21-deficient cells, indicating the essential role of p53 in p21-induced cell cycle arrest. Furthermore, the cells containing p21 were demonstrated to resume proliferation after recovery, in contrast to p21-deficient cells [63]. Several studies have demonstrated that ROS caused a G2/M arrest [65, 66]. In these cases, elevation of ROS was demonstrated to result in p53-independent accumulation of p21, an increase in expression of Chk1, and decrease in Cdc25c. This latter phosphatase causes dephosphorylation of cdc2 under normal conditions and hence an activation of the mitotic cyclin-CDK complexes. This effect of ROS during the G2 phase was accompanied by an increase in both ERK and p38 phosphorylation [67]. Furthermore, the effects of ROS on p21, Chk1, and Cdc25d expression were shown to be dependent on both ERK and p38 activity [67].

Apparently, both cell proliferation and growth arrest have been observed after oxidative stress; the cell response depends on the molecular background of cells and tissues, the location of ROS production, the concentration of individual ROS species, and the antioxidant concentration in the cells [54]. While the growth arrest induced by a little increase of oxidative stress may occur in G1, S, or G2 phases and may be transient, a disproportional increase in intracellular ROS, achieved with cancer chemotherapy, depletion of cells from antioxidant proteins, or generation of ROS by immune cells, can induce a permanent cell cycle arrest, which may end in senescence and apoptosis. Apoptosis is linked to an increase in mitochondrial oxidative stress that causes cytochrome c release and the consequent activation of caspases and cell death [68, 69]. Additionally, superoxide generation through the Rac-1/NADPH oxidase pathway can also induce proapoptotic signalling [70]. Notably, aberrant ROS production by dysfunctional mitochondria has also been shown to increase oxidative-stress-induced cellular senescence [71–73]. Indeed, ROS can induce cellular senescence by activating critical cell cycle sentinels that mediate this process, such as the tumor suppressor proteins p53 and retinoblastoma (Rb) [74]. The signal that originates activation of these checkpoints is the oxidative-stress-induced DNA damage, such as double-strand breaks, and downstream induction of cell cycle regulators p21 and p16 to trigger and maintain senescence/growth arrest [75, 76]. The function mutations of these critical regulators endow cells with the ability for unabated proliferation to the point where the telomeres are critically shortened, a state referred to as “crisis” or mortality stage 2 (M2) where replication ceases [77]. Oxidative stress can also provoke the oxidation of telomeric ends, which are prone to oxidative modification since they are comprised of sequences that contain triple guanine repeats. Oxidation of these repeats makes the telomeric ends more susceptible to breaks and enhances the rate of telomere attrition [78]. Finally, oxidative stress can induce premature senescence by a direct suppression of telomerase activity, mainly by affecting the expression of the catalytic subunit of telomerase (hTERT) [79].

## 4. Biological Effects of HNE in Cancer Cells

As far as it regards the effect of HNE in cancer cells, the majority of research reports indicate that HNE added to cancer cells of diverse origin elicits a reduction of cell proliferation and an induction of apoptosis. The first experiments were done by using cultivated human leukemic cells, which have an undetectable level of lipid peroxidation and do not contain endogenous HNE. In K562 cells, originally derived from a human erythroleukemia, very low concentrations of HNE were found to strongly decrease cell proliferation and to block the expression of the oncogene c-myc, which was highly expressed in untreated cells [80, 81]. Similarly, in human HL-60 leukemia cells, HNE strongly decreases cell proliferation, induces granulocytic-like differentiation, and, at the same time, blocks the expression of the oncogenes c-myc and c-myb [82, 83]. The inhibition of c-myc expression by HNE has also been observed in U937 and ML1 human leukemic cells and in MEL murine erythroleukemic cells, in which HNE induced an onset of differentiation also [84, 85]. These actions of HNE were transient but could be stabilized by a continuous supply of 1 *μ*M HNE, repeated 10–12 times. Moreover, it has been demonstrated, in colon cancer cells, a transitory increase in c-myc expression and a subsequent downregulation, after HNE treatment [86].

In HL-60 human leukemic cells, the blocking of proliferation caused an increase of the proportion of cells in the G0/G1 phase of the cell cycle and a corresponding decrease of S-phase cells. This means that the progression to the S phase of the cycle in this cell line is prevented by HNE treatment [87]. In this cell line, the inhibition of cell cycle progression mostly depends on the inhibition of the cyclin expression, in particular of cyclins D2, D1, and A [88]. The reduced amount of G1 cyclins caused a hypophosphorylation of pRb protein and a consequent blocking of E2F transcriptional activity, also related to the inhibition of E2F4 expression by HNE [89, 90]. The effect of HNE on genes involved in the control of cells cycle progression has been investigated in SK-N-BE neuroblastoma cells. The aldehyde was shown to be able to reduce cell-cycle-related transcriptional activity and to inhibit proliferation in the SK-N-BE neuroblastoma cell line by increasing the expression of p53 family proteins (p53, p63, and p73) and p53 target proteins (p21, bax, and G1 cyclins) [91]. An increase in p53 expression also has been found in germ cells where HNE treatment inhibited proliferation [92]. 

Different research groups demonstrated that HNE inhibited proliferation of human colon tumor cells through regulation of the MAPs kinase pathway [93, 94] or through the PPAR gamma pathway [86]. Moreover, a strong inhibition of cell proliferation was reported also in breast cancer cells (MCF7) treated with conjugated linoleic acid (CLA), which increases the endogenous levels of HNE [95] and in human osteosarcoma cells treated with HNE [96]. In PC3 prostate carcinoma cells, HNE significantly potentiates the antitumor effects of the HDAC inhibitor panobinostat (LBH589). Cell cycle analysis revealed that both single agents and, to a greater extent, their combined treatment induced G2/M arrest. Furthermore, the combination of panobinostat and HNE induced significant DNA damage concomitant with the mitotic arrest [97]. In hepatoma cells, such as 7777 and J42 hepatomas, the inhibitory effects of HNE on cell proliferation are lower, probably due to the presence of a more efficient system removing the aldehydes. Indeed, these cells display a very high expression of aldehyde dehydrogenase 3, that is able to destroy a large amount of the added aldehyde. Its inhibition by antisense oligonucleotide has strong inhibitory effects on cell proliferation, suggesting that this aldehyde plays an important role in this inhibition [98, 99]. In a recent study, performed in SH-SY5Y human neuroblastoma cells, which can be maintained in an undifferentiated state and can be stimulated to differentiate into a neuron-like phenotype in cell culture, the susceptibility to 2,3-dimethoxy-1,4-napthoquinone (DMNQ) and HNE in differentiated and undifferentiated cells has been compared [100]. Results demonstrated that differentiated cells were substantially more resistant to cytotoxicity and mitochondrial dysfunction induced by the reactive lipid species HNE or the reactive oxygen species generator DMNQ and suggest that profound changes in mitochondrial metabolism and antioxidant defenses occur upon differentiation of neuroblastoma cells to a neuron-like phenotype.

In accordance with these observations are the results demonstrating that the effects of HNE on normal cell proliferation were in some cases opposite to that observed in tumor cells. Concerning the atherogenic role of oxidized low-density lipoprotein and the lipid oxidation products, it has been reported that HNE induced vascular smooth muscle cell proliferation [101, 102]. More recently, other authors have shown that the proliferation rate of smooth muscle cells (SMCs) depends on HNE incubation time and concentration: a prolonged treatment with 0.1 *μ*M HNE resulted in an increase of cell growth in young SMC but displayed cytotoxicity in aged SMCs [103]. In the same cell model, Vindis and collaborators [104] demonstrated that short-term incubation of SMCs with oxLDLs and HNE induced platelet-derived growth factor receptor (PDGFR) *β* activation, while long-term incubation triggered a desensitization of PDGFR to its own agonist, with a progressive inhibition of PDGFR-*β* phosphorylation. These authors concluded that PDGFR-*β* is a target for HNE, and its progressive inhibition may contribute to defective SMC proliferation.

A direct comparison between the HNE effect on the growth of human lymphatic leukemia cells and normal human peripheral blood lymphocytes has been done by Semlitsch and collaborators [105], which demonstrated that HNE showed a cytotoxic effect and reduced DNA synthesis in lymphatic leukemia cells, whereas it did not show any significant toxicity on normal lymphocytes. Other important studies compared the gene expression profile, detected after treatment of acute myelogenous leukemia (AML) cells with parthenolide (PTL), with similar signatures in publicly available gene expression profiles deposited into the Gene Expression Omnibus (GEO) [106]. PTL was found to be able to ablate bulk, progenitor, and stem AML cells while causing no appreciable toxicity to normal hematopoietic cells, thus the authors hypothesized that other compounds, able to induce a modification of the gene expression similar to that produced from PTL, could have similar anticancer characteristics. The author found 2 new agents, celastrol and HNE, that had a PTL gene expression signature and effectively eradicated AML at the bulk, progenitor, and stem cell level.

Taken together these last data indicates that HNE strongly reduces the proliferation of tumor cells, but it increases or does not affect proliferation of normal cells in relation to the dose of HNE and the time of exposure. This dual effect may be due not only to the presence of aldehyde-metabolizing enzymes but also to the antioxidant defenses and mitochondrial metabolism.

## 5. Oxidative Stress in Cancer Therapy

**Figure 1:**
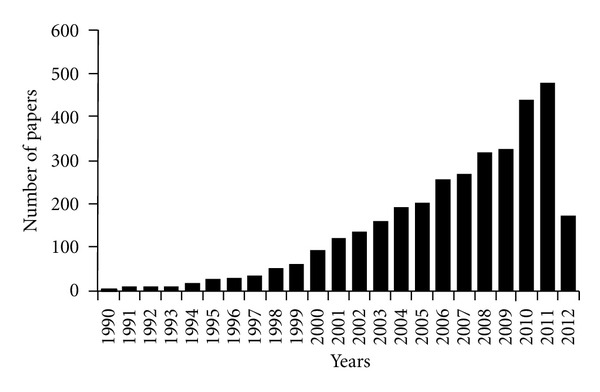
Number of published papers/year, concerning the oxidative stress in cancer therapy.

Several experimental results demonstrated that the increase of ROS in cancer cells may play an important role in the initiation and progression of cancer [107, 108] such that intrinsic oxidative stress is often viewed as an adverse event. However, excessive levels of ROS stress can also be toxic to the cells: cancer cells with increased oxidative stress are likely to be more vulnerable to damage by further ROS insults induced by exogenous agents [109]. Therefore, manipulating ROS levels by redox modulation is a way to selectively kill cancer cells without causing significant toxicity to normal cells [110]. In recent years, an increasing number of experimental results indicated that the increase of ROS is involved in apoptosis induction by chemotherapeutic anticancer agents (Figure 1).

### 5.1. Anticancer ROS-Generating Compounds from Natural Origin

Several ROS inducing compounds, previously investigated as antimicrobial agents, pesticides, or natural products of vegetables, have been demonstrated to possess anticancer activity in a number of cancer models. A naturally occurring ROS-inducing compound, rotenone, a natural hydrophobic pesticide derived from the roots and backs of the *Derris* and *Lonchocarpus* species, has been reported to display anticancer activity through the induction of apoptosis [111–113] in cells derived from human B-cell lymphomas [113], promyelocytic leukemia [114], and neuroblastomas [115]. The increase of ROS caused by rotenone is attributed to irreversible binding and inactivation of complex of the mitochondrial electron transport chain [114]. This can block electron transfer from complex to ubiquinone, resulting in a blockage of the oxidative phosphorylation process and an increase of ROS. In MCF-7 cells, rotenone caused apoptosis through a decrease of the antiapoptotic protein, Bcl-2, and a correspondent increase of the apoptotic protein, Bax. The pharmacological inhibition of JNK and p38 MAPK revealed significant protection against rotenone-induced apoptosis, indicating that the proapoptotic action of rotenone is mediated by these signaling pathways [116].

Cribrostatin 6 is a quinone-containing natural product with antimicrobial activity that has been demonstrated to induce the death of cancer cell lines in culture. Its mechanism of action involves ROS generation, which has been indicated as the primary mechanism of cribrostatin 6-induced apoptosis [117].

D,L-Sulforaphane, a synthetic analogue of naturally occurring L-isomer, abundant in several cruciferous vegetables (e.g., broccoli), is a potent inhibitor of chemically induced cancer in experimental rodents, and it is also known to inhibit growth of human cancer cells in association with cell cycle arrest and reactive oxygen species-dependent apoptosis [118]. Silibinin, a flavonolignan from the seeds and fruits of milk thistle (*Silybum marianum*), is used in the clinic or as dietary supplements against liver toxicity in Asia, Europe, and the USA. Although some evidence indicates an antioxidant activity for silibinin to prevent hepatotoxicity and other pathogenesis of inflammation, ischemia/reperfusion, atherosclerosis, and ageing, recent researches indicate that silibinin induces protective O_2_^•−^ generation in the MCF-7 cell line, and the mitochondrial respiratory chain complexes I, II, and III are involved in O_2_^•−^ formation [119].

Tanshinone IIA, extracted from the dried root of *Salvia miltiorrhiza* (Danshen), is one of the potential candidates undergoing intensive evaluation, despite its traditional role in the treatment of cardiovascular diseases in China. Initial studies have shown that tanshinone IIA exerted cytotoxic effect on a number of human tumor cell lines [120]. More recent studies revealed that induction of apoptosis was the key factor in contributing to the cytotoxic property of tanshinone IIA and that this effect is related to induction of ROS generation [121].

Gallic acid (3,4,5-trihydroxybenzoic acid, GA), a polyhydroxy phenolic compound, is abundant in natural plants such as gallnut, grapes, sumac, oak bark, green tea apple peels, grapes, strawberries, pineapples, bananas, lemons, and in red and white wine. Its antioxidative DNA-damage action has been well documented [122]. However, gallic acid induces apoptosis in several cancer cell lines by increasing ROS level and GSH depletion [123].

Phx-3 (2-aminophenoxazine-3-one) is an oxidative phenoxazine, like actinomycin D, synthesized by the reactions of o-aminophenols with bovine hemoglobin. Phx-3 exerts anticancer activity against various cancer cells “*in vitro*” and “*in vivo*,” promoting both caspase-dependent and caspase-independent apoptosis [124]. Moreover, Zheng et al. (2010) reported that Phx-3 might be a strong anticancer drug against lung cancer, which is intractable to chemotherapy, by causing various early events, including the decrease of pHi and ROS production, and finally inducing cellular apoptosis [125].

Anthocyanidins, a subclass of flavonoids, have been suggested as useful agents for chemotherapy since they can trigger apoptosis in human leukemia cell lines through induction of oxidative stress [126]. Recently, it has been demonstrated that cyanidin and delphinidin induced apoptosis preferentially in drug-resistant LoVo/ADR metastatic colon cancer cells. This action was accompanied by ROS accumulation, inhibition of glutathione reductase, and depletion of GSH [127].

The antitumor agent sesquiterpene lactone parthenolide shows several biological activities, including inhibition of NF-kB and STAT3 DNA-binding activity, inhibition of MAP kinase activity, induction of oxidative stress, and reduction of GSH, followed by G2/M arrest and apoptosis. It is the active ingredient of the herb feverfew (*Tanacetum parthenium*), which is used orally as a migraine prophylaxis, and for arthritis, fever, and stomachache. Besides its anti-inflammatory and antimigraine properties, parthenolide also shows anticancer activities in a variety of cell lines [128]. Parthenolide modulates multiple targets, thereby contributing to its various “*in vitro*” and “*in vivo* effects.” Nakshatri et al. reported that parthenolide reverses resistance of breast cancer cells to tumor necrosis factor-related apoptosis-inducing ligand through sustained activation of c-Jun N-terminal kinase [129]. Other authors have demonstrated that parthenolide induces a robust apoptosis of primary stem and progenitor acute myeloid leukemia cells thorough the induction of oxidative stress and inhibits ion of NF-*κ*B [130]. Inhibition of NF-*κ*B activity, constitutive in many types of cancers, via either interaction with IKK or more directly with the p65 subunit of NF-*κ*B, is considered one of the main mechanisms of its action. At the epigenetic level, parthenolide reduces HDAC1 level and, by inhibiting DNMT2 activity, induces global hypomethylation of DNA, which can restore the expressions of some suppressor genes [128]. A unique property of parthenolide is its ability to induce cell death mainly in cancer cells, while sparing healthy ones, and it also protects normal cells from UVB and oxidative stress [128].

Butein, a bioactive flavonoid isolated from numerous native plants, has been shown to induce apoptosis in human cancer cells. Recently, it has been reported that the treatment of neuro-2A neuroblastoma cells with butein decreased the viability through the increase of intracellular ROS levels and reduction of the Bcl-2/Bax ratio, triggering the cleavage of procaspase 3 and poly(ADP-ribose) polymerase (PARP). Moreover, the pretreatment with the antioxidant agent, N-acetyl cysteine, blocks butein-induced ROS generation and cell death [131].

Romidepsin, isolated from *Chromobacterium violaceum* (previously called depsipeptide, Kf228, Fr901228), is the second histone deacetylase inhibitor (HDACi) approved for the treatment of advanced stages of cutaneous T-cell lymphoma [132]. HDAC inhibitors lead to a wide spectrum of biological effects, including induction of apoptosis inhibition of angiogenesis, induction of cellular senescence, and induction of oxidative stress caused by mitochondrial injury. In particular, for romidepsin, it has been reported that its apoptotic action is linked to the generation of hydrogen peroxide and is prevented by N-acetyl-cysteine, thus suggesting that reactive oxygen species participate in apoptosis [133].

### 5.2. Drugs Currently Used in Chemotherapy Acting by ROS Generation

Many antitumor agents, such as vinblastine, cisplatin, mitomycin C, doxorubicin, camptothecin, inostamycin, and neocarzinostatin, exhibit antitumor activity via ROS-dependent activation of apoptotic cell death, suggesting potential use of ROS as an antitumor agent. Thus, a unique anticancer strategy named “oxidation therapy” has been developed by inducing cytotoxic oxystress for cancer treatment.

Vinblastine belongs to the class of vinca alkaloids which includes vinorelbine, vincristine, vinblastine, and vindesine. These substances bind to tubulin as potent inhibitors of mitotic microtubule polymerization. It has been demonstrated that vinca alkaloid sequentially induced mitochondrial transmembrane potential loss and caspase-dependent apoptosis. The role played by ROS seems to be essential in vinca alkaloid-induced aberrant JNK-mediated Mcl-1 downregulation and DNA damage followed by mitochondrial dysfunction-related apoptosis but not in determining mitotic arrest [134].

Cisplatin is one of the most commonly used cytotoxic agents in the treatment of a variety of solid malignant tumors, for example, in the head and neck, lungs, ovary, bladder, and testicles. Although treatment with this drug is often effective, serious side effects such as nausea, nephrotoxicity, neurotoxicity, and ototoxicity occur often. Several mechanisms are believed to mediate cisplatin-induced apoptosis. The “traditional” mechanism involves covalent binding of cisplatin to guanine bases on DNA; the formation of inter- and intrastrand chain cross-linking; induction of p53, cell cycle arrest, and apoptosis. More recently, it has been shown that ROS generated by cisplatin could increase lipid peroxidation, which alters enzymes and structural proteins and directs the cell to an apoptotic pathway. Also, cisplatin-induced apoptosis could involve the inflammatory pathway [135].

Mitomycin C has been shown to induce apoptosis through DNA strand breakage and ROS induction [136]. Mitomycin C has been used in treating a wide variety of cancers including ovarian cancer where it showed activity in phase II trials. Recently, it has been reported that tetrathiomolybdate, an anticancer agent acting through the inhibition of cellular antioxidant copper zinc superoxide dismutase (SOD1) and through elevation of levels of cellular ROS, increases the apoptosis induced by mitomycin C action in ovarian cancer cells. The increased cytotoxicity was correlated with the activity of ROS, loss of a prosurvival factor, and the appearance of a proapoptotic marker [137].

Doxorubicin, an anthracycline antibiotic and topoisomerase inhibitor, is one of the most widely used and successful antitumor drugs, but its cumulative and dose-dependent cardiac toxicity has been a major concern of oncologists in cancer therapeutic practice for decades [138]. The toxicity toward cardiomyocytes and the induction of apoptosis in cancer cells are mediated by the direct oxidative DNA damage leading to indirect H_2_O_2_ generation through PARP and NAD(P)H oxidase activation. DOX caused site-specific oxidative DNA damage in the presence of copper(II), which may contribute to apoptosis [139].

Camptothecin, an inhibitor of DNA topoisomerases, is a plant alkaloid first identified from the Chinese tree, *Camptotheca acuminata*. Camptothecin acts on cancer cells by inhibiting topoisomerase1, an enzyme that covalently links the tyrosine to the DNA phosphate at the 3′-end of the broken DNA while generating a 5′-hydroxyl end at the other end of the break. Two water-soluble camptothecin derivatives are presently approved by the FDA for IV administration: topotecan and irinotecan. Topotecan (Hycamtin) is used to treat ovarian cancers and small-cell lung cancers. However, hematological toxicity is a common side effect due to the destruction of bone marrow progenitors [140]. Camptothecin as well as inhibitors of topoisomerases induces breaks in the DNA that ultimately lead to programmed cell death. However, it has been demonstrated that ROS production is involved in camptothecin-induced apoptosis. In camptothecin-treated DAOY medulloblastoma, cell-reactive oxygen species, especially O_2_, were elevated, and the antioxidants glutathione and N-acetyl-cysteine prevented cell death [141]. 

Other new ROS-inducing chemicals are under examination as promising anticancer drugs, such as a naturally occurring ROS-inducing compound, beta-phenyl beta-phenyl ethyl isothiocyanate, which selectively killed cancer cells but not normal cells [142]. Another promising compound, NSC-741909, induces robust ROS generation in sensitive but not in resistant cancer cells [143]. Celastrol, a triterpene, shows in acute myeloid cells a pattern of action similar to that detected for parthenolide and HNE [106]. It facilitates TRAIL-induced apoptosis by downregulating cell survival proteins and upregulating death receptors [144]. These molecules have the potential to be effective for all cancer cells, although ROS induction through anticancer drugs is discretely studied and limited to only specific type of cancer cells. Initial ROS induction seems to be common for many anticancer agents for most of the cancers.

## 6. Combined Anticancer Therapies: Therapy with ROS-Inducing Agents

Besides the intrinsic ROS-generating activity of many anticancer drugs, there is evidence demonstrating an increase of apoptotic activity when the anticancer drugs were used in association or in combined treatments with ROS-inducing agents. In both cases, the cell death was accompanied by an increase of intracellular ROS. Most experiments have been performed in particularly aggressive malignance or in cancers poorly affected by conventional chemotherapy. Some studies have been performed in pancreatic cancer, which is one of the most lethal digestive system malignancies and has a very poor prognosis. In pancreatic cancer cells treated with MDA-7/IL24 and an ROS inducer (arsenic trioxide or dithiophene), an increase of apoptosis has been demonstrated. This effect could be reversed by ROS inhibitors such as N-acetyl-L-cysteine implying the role of ROS in inducing cell death in these cells [145]. Another study demonstrated that the treatment with dihydroartemisinin, a semisynthetic derivative of artemisinin, with antitumor activity and Apo2 ligand or tumor necrosis factor-related apoptosis-inducing ligand (Apo2L/TRAIL) increased the apoptotic effect in human pancreatic cancer cells through reactive oxygen species-mediated upregulation of DR5 [146]. In pancreatic and hepatoma cell lines, the use of sorafenib, an inhibitor of Raf family kinases, and vorinostat, a histone deacetylase inhibitor, interacts in a synergistic fashion to kill carcinoma cells by activating CD95. Moreover, low doses of sorafenib and vorinostat, but not the individual drugs, rapidly increased ROS, Ca^2+^, and ceramide levels in gastrointestinal tumor cells. The authors demonstrated that induction of cytosolic Ca^2+^ by sorafenib and vorinostat is a primary event that elevates dihydroceramide levels, each is an essential step in ROS generation that promotes CD95 activation [147]. Another histone deacetylase inhibitor, trichostatin A, in combined treatment with gemcitabine, a nucleoside analogue of cytidine (2′,2′-difluorodeoxycytidine; dFdC) widely used chemotherapeutic regimes for the treatment of pancreatic cancer, showed a synergistic effect in inhibiting growth of four pancreatic adenocarcinoma cell lines and in inducing apoptosis. This effect was associated with the induction of ROS by gemcitabine, increased expression of the proapoptotic BIM gene by both trichostatin A and gemcitabine, and downregulation of the 5′-nucleotidase UMPH type II gene by trichostatin A [148]. In a panel of 11 different cell lines arising from prostate, breast, lung, colon, cervix, bladder, and kidney cancers, a combination therapy of As_2_O_3_ with L-buthionine-sulfoximine (BSO), which inhibits a critical step in glutathione synthesis, effectively enhanced the “*in vitro*” growth inhibition effect of As_2_O_3_ alone. Biochemical analysis showed that combined use of As_2_O_3_ and BSO blocked H_2_O_2_-scavenging systems including glutathione, catalase, and glutathione peroxidase, and that the degree of this blockade was well correlated with intracellular ROS levels and sensitivity to this treatment [149]. In eight neuroblastoma cell lines, the increased inhibition of cell growth induced by combination of MK-2206, a novel allosteric Akt inhibitor, and rapamycin was mediated by ROS production [150]. Similarly, in this cell model, the use of BSO increased the cytotoxic effects of fenretinide, in neuroblastoma monolayers and spheroids and in ROS-producing cell lines [151]. In the treatment of hepatocellular carcinoma, the single agent As_2_O_3_ was inefficient for phase II trials, demonstrating that new modalities of treatment with enhanced therapeutic effect are needed. In a recent study, Chen et al. [152] showed that oridonin, a diterpenoid isolated from traditional Chinese medicine rabdosia rubescens, greatly potentiated apoptosis induced by As_2_O_3_ in hepatocellular carcinoma cells. Moreover, the two-drug combination enhanced tumor suppression activity in the murine hepatocellular carcinoma model compared with single-agent treatment “*in vivo.*” The synergistic proapoptotic effect of the combined treatment leads to an increase in the intracellular reactive oxygen species level, and N-acetyl-L-cysteine completely blocks this effect. The combination treatment induced an ROS-dependent decrease in mitochondrial membrane potential and relocation of Bax and cytochrome C. In addition, the cotreatment of oridonin and As_2_O_3_ induced ROS-mediated downregulation of Akt and XIAP and inhibition of NF-*κ*B activation [152]. To treat hepatocellular carcinoma cells, in association with As_2_O_3_, Poly I : C (polyinosinic acid : polycytidylic acid), an analogue of dsRNA (double-stranded RNA [153], genistein [154], and ascorbic acid [155]), has been previously used. In all cases, a synergistic effect has been observed in inducing apoptosis, in reducing cell proliferation, and in increasing intracellular ROS concentration.

In leukemia cells, several reports indicate that combined therapy, involving the intracellular increase of ROS, can augment the apoptosis induction. As far as it regards nonsteroidal anti-inflammatory drugs (NSAIDs), it has been reported that diclofenac induces growth inhibition and apoptosis of HL-60 cells through modulation of mitochondrial functions regulated by ROS. ROS generation occurs in an early stage of diclofenac-induced apoptosis preceding cytochrome c release, caspase activation, and DNA fragmentation. N-Acetyl-L-cysteine suppresses ROS generation, Akt inactivation, caspase-8 activation, and DNA fragmentation. The combined treatment with 2-methoxyestradiol, a superoxide dismutase inhibitor, significantly enhances diclofenac-induced apoptosis [156].

In another class of drugs, the histone deacetylase inhibitors coadministrated together the alkyl-lysophospholipid perifosine induce mitochondrial dysfunction (cytochrome c and apoptosis-inducing factor release), caspase-3 and -8 activation, apoptosis, and a marked decrease in cell growth in U937 as well as HL-60 and Jurkat leukemia cells. These events are associated with inactivation of extracellular signal-regulated kinase (ERK) 1/2 and Akt, p46 c-jun-NH2-kinase (JNK) activation, and a pronounced increase in generation of ceramide and ROS [157]. According to these results, the treatment with the multiple receptor tyrosine kinase inhibitor, AEE788, together with the histone deacetylase inhibitors (LBH589, LAQ824, and trichostatin A) results in synergistic induction of apoptosis in non-small-cell lung cancer (MV522, A549), ovarian cancer (SKOV-3), and leukemia (K562, Jurkat, and ML-1) cells and in OV202hp cisplatin-resistant human ovarian cancer cells. AEE788 alone or in combination with LBH589 inactivated mitogen-activated protein kinase (MAPK) and Akt cascades. Increased apoptosis is correlated with enhanced ROS generation. The free radical scavenger N-acetyl-l-cysteine not only substantially suppressed the ROS accumulation but also blocked the induction of apoptosis mediated by cotreatment with AEE788 and LBH589 [158]. A novel therapy against acute myeloid leukaemia (AML) has been proposed for the patients over sixty years of age by using the lipid-regulating drug bezafibrate and the sex hormone medroxyprogesterone acetate. These compounds showed a potent antileukaemic activity against AML cell lines and primary AML cells. Combined treatment of AML cells with bezafibrate and medroxyprogesterone acetate elevated ROS and delivered the antineoplastic actions of 15d-prostaglandin J [159].

Niclosamide is a Food and Drug Administration-approved antihelminthic agent, which inhibits the transcription and DNA binding of NF-*κ*B in acute myelogenous leukemia. It blocked tumor necrosis factor-induced I*κ*B alpha phosphorylation, translocation of p65, and expression of NF-*κ*B-regulated genes. Combined treatment with niclosamide and cytarabine, etoposide, and daunorubicin shows synergistic activity in inducing apoptosis and in increasing the ROS level in acute myelogenous leukemia [160].

In peripheral blood mononuclear cells, isolated from chemorefractory patients with chronic lymphocytic leukemia, the efficacy and mechanism of action of cisplatinum in association with fludarabine have been investigated [161]. The two compounds act synergistically in inducing apoptosis. The response involved generation of ROS, which led to specific upregulation of the proapoptotic BH3-only protein Noxa.

Taken together, these results demonstrated that in multiple cancer models an increase of oxidative stress leads to an increase of apoptosis through mechanisms different in relation to the cell type and the anticancer used. However, a common feature in all cancers treated with these drug combinations is the increase of intracellular ROS.

## 7. Contribution of Lipid Peroxidation Products in Cancer Treatments

Among the works cited above, which demonstrate the ROS induction during the treatment with chemotherapeutic agents, only a small number of reports take into account the role played by the lipid peroxidation product originated from ROS induction. Important evidence is reported by Sturlan et al. [162], who demonstrated that some polyunsaturated fatty acids, such as docosahexaenoic acid (DHA), can sensitize various tumor cells to ROS-inducing anticancer agents. Apoptotic cell death was preceded by collapse of the mitochondrial membrane potential, increased expression of Bax, and caspase-3 activation. The combined effect of As_2_O_3_ and DHA was associated with increased production of intracellular ROS and toxic lipid peroxidation products and was abolished by the antioxidant vitamin E or when oleic acid (a nonperoxidizable fatty acid) was used in place of DHA. The authors conclude that ROS reduction and toxic lipid peroxidation products constitute the key mediators contributing to the combined effect of As_2_O_3_ and DHA. Similar results were obtained in 12 different tumor cell lines, including MDA-MB-468, SK-BR-3, MCF-7 (breast cancer), ES-2, SKOV-3 (ovarian cancer), HT-29, SW-620, LS-174T (colon cancer), PC-3 (prostate cancer), HeLa (cervical cancer), PANC-1 (pancreatic cancer), and one primary melanoma cell line resistant to treatment with either As_2_O_3_ or DHA alone. These cells, when exposed to As_2_O_3_ and DHA, showed an induction of apoptosis and a concomitant increase of intracellular lipid peroxidation products. Moreover, the combined effect of As_2_O_3_ and DHA was selectively toxic for malignant cells since no cytotoxic effect was observed in normal skin fibroblasts, human microvascular endothelial cells, and peripheral blood mononuclear cells derived from healthy donors [163]. The toxic effect of the association of As_2_O_3_ and DHA is also observed when other polyunsaturated fatty acids (PUFAs) (i.e., eicosapentaenoic acid, arachidonic acid, and gamma-linolenic acid) were associated to As_2_O_3_ in the treatment of fourteen tumor cell lines. Twelve of 14 As_2_O_3_-resistant cell lines tested were resistant to PUFA monotherapy. However, combined treatment with As_2_O_3_ and either PUFA significantly reduced cell viability in a dose-dependent manner with AA being the most potent As_2_O_3_ enhancer. The combined cytotoxic effect of combined treatment was due to induction of apoptosis, preceded by increased intracellular aldehyde products of lipid peroxidation and was abolished by the antioxidant vitamin E. These effects were selectively toxic for malignant cells, and no cytotoxic effect was observed in normal skin fibroblasts and human microvascular endothelial cells [164].

A potent antileukaemic activity has been observed by using the combination of the lipid-regulating drug bezafibrate and the sex hormone medroxyprogesterone acetate against AML cell lines and primary AML cells. It has been reported that, beside the ROS increase, an increase of lipid peroxidation products also occurs [159].

In colon cancer cells, the combined treatment with 5-fluorouracil and resveratrol (trans-3, 4′, 5-trihydroxystilbene) results in a significant decrease in long-term cell survival and an imbalance in cellular antioxidant activities, leading to a significant increase in the levels of both intracellular ROS and lipid peroxides. Combined treatment with resveratrol sensitizes colon cancer cells to 5-fluorouracil, inducing a further increase in oxidative stress, which was linked to the inhibition of AKT and STAT3 proteins, which are known to have oncogenic potential in colorectal carcinomas [165]. A paramount role of lipid peroxidation of PUFAs not only in carcinogenesis but also in potential therapeutic strategy on colorectal cancer has been recently outlined in the review by Cai et al. [166]. The authors conclude that since the end products of lipid peroxidation, such as malondialdehyde and HNE, have a cytotoxic effect not only in normal cells but also in cancer cells, PUFAs may play a potential role in colorectal cancer treatment.

Our research group recently demonstrated that HNE significantly potentiates the antitumor effects of the HDAC inhibitor panobinostat (LBH589) in the PC3 prostate cancer cell model. Panobinostat and HNE inhibit proliferation of PC3 cells, and the combination of the two agents results in a significant G2/M arrest and cell death which occurs after the combined treatment only. Furthermore, HNE and, to a greater extent, the combined treatment induce dephosphorylation of Cdc2 leading to progression into mitosis. Moreover, our results showed that the combination of panobinostat and HNE induced significant DNA damage concomitant with the mitotic arrest [167]. Previously, we demonstrated that PPAR-alpha ligands (clofibrate and ciprofibrate) and PPAR-gamma ligands (troglitazone and 15d-prostaglandin J2) inhibit growth and induce monocytic differentiation in HL-60 cells, and HNE, which alone induces granulocytic-like differentiation of HL-60 cells, potentiates the monocytic differentiation induced by ciprofibrate, troglitazone, and 15d-prostaglandin J2. Moreover, HNE treatment significantly inhibits U937 cell growth and potentiates the inhibition of cell growth in PPAR-gamma ligand-treated cells [168].

## 8. Radiation Therapy, ROS Generation, and Lipid Peroxidation Induction

The most important physical stimulus that causes ROS is ionizing radiation (IR). IR is one of the main treatment modalities used in the management of cancer, and it can evoke a series of biochemical events inside the cell. These events comprise many important cellular processes, such as DNA damage and repair, apoptosis, cell cycle control, signal transduction, and oxidative stress response [169]. The IR effects include damage of DNA, lipids, and proteins by the energy of radiation, and by the ROS derived from intercellular water. The secondary response involved signal transduction and gene expression. Among these effects, ROS induced by IR are crucial in inducing cell death. The mechanisms of ROS induced by IR involved in apoptotic cell death and cell cycle arrest have been widely investigated in the field of cancer radiotherapy. The response to radiation depends on the type and dose of radiation, tissue sensitivity, and intracellular factors that include position in the cell cycle, oxygen concentration, and levels of antioxidants. Radiation in the presence of high oxygen concentration increases the amount of oxygen free radicals and makes free radical-induced damage permanent. In the absence of oxygen, damage produced by the indirect action can be repaired. Oxygen modifies the indirect, but not the direct action of radiation. Antioxidants reduce free radicals and repair damage, maintaining integrity of normal cells and affecting tumor cells to a lesser extent [170].

The deleterious cellular effects by ROS include DNA damage and membrane oxidative damage. The formation of single- and/or double-stranded breaks of DNA resulted in cell cycle arrest and recruitment of DNA repair enzymes to rescue cells from the damage. Beyond DNA damage, another major target of radiation and ROS is believed to be the membranes of cytoplasmic organelles and plasma membrane of cells. It is observed that membrane lipids are easily peroxidised by ROS produced by IR, causing structural and functional impairment [171–174]. Oxidative damage leads to an alteration in the both lipid bilayer fluidity and permeability properties. In the context of cellular response to radiation, the contributions of radiation oxidative damage to membranes as well as to DNA damage via ROS appears rather complex, and these initiators pathways of cytotoxicity seem to be intimately linked in the development of radiation induced cell death. Moreover, lipid peroxidation induced by IR leads to the production of aldehydes which, in turn, may contribute to apoptosis induction and cell cycle arrest. 

Although the presence of reactive aldehydes derived from lipid peroxidation after IR has been long demonstrated, a few studies have investigated the role played by these end products in inducing IR effects. In prostate cells, the relationship between IR treatment, ROS production, lipid peroxidation, glutathione (GSH) levels, and DNA damage of the human benign prostate hyperplasia BPH-1 cell line, and two prostate cancer cell lines, LNCaP, which is androgen sensitive, and DU-145, which is androgen nonresponsive, has been investigated [175]. The authors demonstrated that DU-145 cells were characterized by higher DNA damage, more evident extent of lipid peroxidation, and lower levels of ROS and GSH compared to BPH-1 or LNCaP. These results could suggest that the increase of lipid peroxidation, more than the ROS production, is responsible for DNA damage. Another recent study in PC3 prostate cancer cells injected into nude BALB/c mice demonstrated that the radiotherapy efficacy of prostate cancer can be increased with pro-oxidant, and the reduction of tumor size is proportional with the increase of lipid peroxidation [176].

By using docosahexaenoic acid (DHA), a 22-carbon omega-3 PUFA with 6 cisdouble bonds highly susceptible to peroxidation induced by oxidative stress, in association with IR, Kikawa et al. demonstrated a decrease in cell proliferation and an induction of cell death accompanied by an increase of lipid peroxidation in A549 lung adenocarcinoma cells [177]. In the same cell model, Dittmann et al. found that the radiation-induced src activation and EGFR stabilization can be mimicked by addition of HNE. The radiation-generated HNE is bound to EGFR and src and correlated with complex formation following radiation [178]. The authors suggest a scenario, where radiation-induced lipid peroxidation results in generation of HNE. HNE activates the redox-sensitive switch of src, which results in a conformational alteration associated with increased kinase activity. Activated src phosphorylates EGFR at Y845 and caveolin 1 at Y14, which leads to internalization of EGFR into caveolae and transports into nucleus. Nuclear EGFR regulates DNA-PK activity and supports DNA-repair processes [179].

In diverse cell models such as Ehrlich ascites, human cervical, and breast cancer cells, the cytotoxic effects of phytochemicals which increase lipid peroxidation, in combination with ionizing radiation, have been demonstrated [180], suggesting that modulation of membrane peroxidative damage and intracellular ROS may help achieve efficient killing of cancer cells which may provide a new approach to developing effective treatment of cancer. According to these results, other authors demonstrated that the addition of omega-3 PUFAs can enhance the radiosensitivity of LS174T, CO112, and Caco-2 colon cancer cells, in terms of cell growth, survival, and apoptosis [181]. The addition of omega-3 PUFAs induced a dose-dependent additive decrease in cell survival after irradiation via apoptosis induction. Moreover, the formation of lipid peroxidation products and modulation of cyclooxygenase II activity seemed to be involved, because coincubation with vitamin E abolished the effect. 

## 9. Oxidative Stress and Lipid Peroxidation Products in Cancer Chemo- and Radioresistance

**Figure 2:**
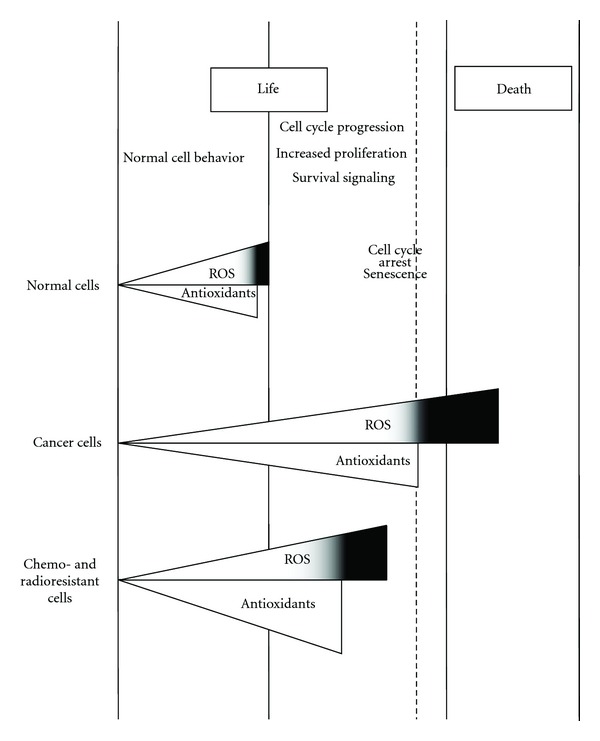
ROS level in normal, cancer, and chemo- and radio-resistant cancer cells. Under physiological conditions, normal cells maintain redox homeostasis with a low level of basal ROS by controlling the balance between ROS generation (pro-oxidants) and elimination (antioxidant capacity). Normal cells can tolerate a certain level of exogenous ROS stress owing to their “reserve antioxidant capacity.” The antioxidant reserve can prevent cell transformation and cell death. In cancer cells, the increase in ROS generation from metabolic abnormalities and oncogenic signalling may trigger a redox adaptation response, leading to an upregulation of antioxidant capacity, high ROS generation, and elimination to maintain the ROS levels below the toxic threshold. A further increase of ROS stress and in lipid peroxidation products in cancer cells (black space) using exogenous ROS-modulating agents or lipid peroxidation substrates is likely to cause elevation of ROS above the threshold level, leading to cell death. This might constitute a biochemical basis to design therapeutic strategies to selectively kill cancer cells using ROS-mediated mechanisms. Finally, excessive increase in intracellular ROS levels (and in lipid peroxidation products) as mediated by radiation therapy and chemotherapeutics may be repulsed by the tumour cells through an increase in the expression of endogenous antioxidants. This redox adaptation not only enables the cancer cells to survive under increased ROS stress, but also provides a mechanism of resistance to many anticancer agents and radiation therapy.

Although the idea of inducing preferential cancer cell death by an ROS-mediated mechanism showed promising therapeutic activity in experimental and clinical studies, some cancer cells, especially those in advanced disease stages, have become highly adapted to intrinsic oxidative stress with upregulated antioxidant capacity. This redox adaptation not only enables the cancer cells to survive under increased ROS stress, but also provides a mechanism of resistance to many anticancer agents and radiation therapy [182]. It has been demonstrated that preexposure of normal epithelial cells to low but continuous levels of exogenous oxidants confers cellular resistance to subsequent oxidative stress even at a higher level [183]. Since cancer cells actively produce high levels of ROS and are consistently exposed to such endogenous oxidants, it is possible that the intrinsic oxidative stress may exert a selective pressure and increase the population of cells that are capable of stress adaptation [184, 185]. Those cells that survive oxidative stress are likely to have acquired adaptive mechanisms to counteract the potential toxic effects of elevated ROS and to promote cell-survival pathways [186] (Figure 2). Additionally, the generation of endogenous ROS in combination with the drugs which initially induces ROS production may contribute to a decrease in the sensitivity to these drugs in long-term treated cancer cells. Indeed, it has been observed that cisplatin or chlorambucil, which initially induces ROS production in ovarian carcinoma A2780 cells, after prolonged drug treatment with either drug actually reduces the ROS level making those cells resistant to the drug [187, 188]. Thus, a decrease of ROS level in prolonged drug-treated cells is not a secondary cellular outcome; instead, it is a primary mechanism for specified drug resistance.

Chemoresistance and radioresistance in cancer are the major obstacles to successful application of cancer therapy. Elevation of certain transcription factors, antioxidants, and survival signals as a result of redox adaptation probably explains the drug-resistant phenotype [189, 190]. Moreover, the inflammatory tumor microenvironment can modulate not only cancer development but also cancer responsiveness and resistance to conventional anticancer therapies [191].

Experimental studies have led to the identification of various cancer cell-intrinsic resistance mechanisms, for example, activation and/or overexpression of drug transporter proteins (e.g., P-glycoprotein), altered expression of detoxifying enzymes (e.g., glutathione S-transferase), or resistance to apoptosis/senescence pathways [192–195]. In addition, several chemotherapeutic agents, such as paclitaxel, vinblastine, vincristine, doxorubicin, daunomycin, 5-fluorouracil, cisplatin, and tamoxifen, have also been shown to activate the transcription factor NF-*κ*B [196–198]. The NF-*κ*B is one of the major chemoresistance-related antiapoptotic factors. Many human cancers possess high levels of the constitutive NF*κ*B activity, which can be further induced by some anticancer drugs. High NF*κ*B activity links inflammation and tumourigenesis [199]. Activated NF-*κ*B triggers a series of molecular reactions including upregulation of antiapoptotic protein-encoding genes [200] that induce cancer chemoresistance. High NF-*κ*B activity has been identified in drug-resistant cancer cells, and ectopic overexpression of NF-*κ*B can block anticancer drug-induced apoptosis [201]. Overexpression of p50 and p65, the two subunits of NF-*κ*B, results in increased NF-*κ*B activity and induces 5-fluorouracil and gemcitabine resistance [201, 202].

In most cancers, cell resistance is associated with activation of prosurvival pathways, such as NF-E2-related factor 2 (Nrf2) [203] or elevated glutathione (GSH) levels [204]. For example, some studies demonstrated that in DOX-resistant human colon cancer cells (HT-29-DX), the level of GSH is higher than that in HT-29 chemosensitive cells [205].

Furthermore, recent work suggests that the transcription factor Nrf2 is responsible for resistance to the GSH-depleting agent BSO, and abrogation of Nrf2 in combination with BSO seemed to be an effective therapeutic strategy [206].

Nrf2 protein serves as a sensor of chemical- and radiation-induced oxidative and electrophilic stress [207]. Nrf2 resides predominantly in the cytoplasm where it interacts with actin-associated cytosolic protein, Keap1 (Kelch-like ECH-associated protein 1, also known as Nrf2 inhibitor, INrf2), which inhibits the Nrf2 activity. Under oxidative stress, Keap1 releases Nrf2 which translocates in the nucleus. The mechanisms by which Nrf2 is released from Keap1 under stress have been actively investigated. A consensus has emerged that oxidative/electrophilic stress modification of Keap1Cys-151 followed by PKC*δ*-mediated phosphorylation of Nrf2Ser-40 leads to the release and stabilization of Nrf2 [207]. In the nucleus, Nrf2 coordinately activates the transcription of a battery of cytoprotective proteins leading to reduced apoptosis, enhanced cell survival, and increased drug resistance. This is achieved through Nrf2 binding to antioxidant response element (ARE) present in the promoter regions of cytoprotective genes including glutathione S-transferases [208]. The overexpression of glutathione S-transferases (GSTs), enzymes that catalyze the conjugation of reduced glutathione to electrophilic compounds, may reduce the reactivity of various anticancer drugs [209]. GSTs are also responsible for the resistance toward HNE injury after treatment with butyrate in colon cancer cells [210].

Several reports indicate that Nrf2 activity increases the chemoresistance of cancer cells. Lau et al. demonstrated a strong positive correlation between Nrf2 levels and resistance of three cancer cell lines to chemotherapeutic drugs such as cisplatin, doxorubicin, and etoposide [211]. According to these findings, the role of Nrf2 in determining the efficacy of cisplatin was also demonstrated in ovarian cancer cells using small interfering RNA knockdown of Nrf2 [212]. Moreover, the stable overexpression of Nrf2 resulted in enhanced resistance of cancer cells to chemotherapeutic agents including cisplatin, doxorubicin, and etoposide. Inversely, downregulation of the Nrf2-dependent response by overexpression of Keap1 or transient transfection of Nrf2-small interfering RNA (siRNA) rendered cancer cells more susceptible to these drugs [213]. Downregulation of Keap1, consequent to gene mutations or loss of heterozygosity in the Keap1 locus, has been identified in lung cancer cell lines or cancer tissues and results in an upregulation of Nrf2 and in transactivation of its downstream genes [214]. We recently demonstrated that increased Nrf2 activity resulted in a reduction of HNE sensitivity in prostate cancer cells and that the inhibition of Nrf2 expression with specific siRNA resulted in a reduction in GST A4 expression and GS-HNE formation, indicating that Nrf2 controls HNE metabolism [215].

Nrf2 is also involved in radioresistance. Indeed, ionizing radiation activates the Nrf2 antioxidant response [216], and it has been established that the constitutive activation of Nrf2 protects against ionizing radiation toxicity and confers radioresistance in mouse embryonic fibroblasts [217]. In a recent work, Lee et al. demonstrated that the inhibition of Nrf2-binding activity and expression by 4-(2-Cyclohexylethoxy aniline, IM3829) inhibit the increase of the Nrf2 target genes induced by treatment with tertiary butylhydroquinone or radiation. Combined treatment with IM3829 and radiation significantly inhibited clonogenic survival of H1299, A549, and H460 lung cancer cells, suggesting that the blocking of Nrf2-dependent antioxidant responses could be a promising strategy for increasing the radiosensitivity of human lung cancer [218].

Other mechanisms involved in the drug resistance regard the transport of xenobiotics and their metabolites by ATP-binding cassette (ABC) transporters particularly P-glycoprotein (Pgp) and the multidrug resistance-associated protein (MRP1), which have been extensively studied during the last decade [219]. A link between Nrf2, multidrug-resistant proteins (MRPs), and HNE has been demonstrated by Mahaffey et al. [220]. MRPs can be increased by chemotherapy, radiation, and other xenobiotic stresses although the underlying mechanism remains largely unknown. Since HNE has been established to cause the activation of the Nrf2-EpRE signaling and cytoprotective gene induction [221] and the MRP3 induction was dependent upon the transcription factor Nrf2 [220], the HNE upregulation of MRP3 mRNA and protein levels in cell lines with wild-type Keap1, but not in the Keap1-mutant NSCLC cell lines, supports the hypothesis that MRP3 induction by HNE involves Nrf2 activation. Another mechanism of chemo- and radioresistance involves RLIP76. It has been demonstrated that the protein RLIP76, which is a nonABC, GTPase-activating protein, functions as an alternative transporter of the end products of detoxification pathways such as glutathione conjugates and xenobiotics. RLIP76 may act in parallel with ABC transporters, or it may be the predominant pump in certain cell types or situations [222]. It has been suggested that RLIP76 plays a major role in the mechanisms of drug resistance [223]. Indeed, in K562 human myelogenous leukemia cells, RLIP76 overexpression confers a broad resistance to multiple chemotherapy drugs including cisplatin, melphalan, doxorubicin, daunorubicin, vincristine, vinblastine, vinorelbine, and mitomycin-C [224]. Conversely, inhibition of RLIP76 by polyclonal antibodies causes increased drug accumulation and increased cytotoxicity. RLIP76 plays an important role in the transport of HNE, too. Sharma et al. demonstrated that RLIP76 mediates active transport of GS-HNE, according to previous results showing that RLIP76-mediated efflux of GS-HNE regulated the intracellular concentration of HNE and thereby affected HNE-mediated signaling [225]. The role of RLIP76 in radioresistance has been outlined by the study in RLIP76(−/−) and RLIP76(+/+) C57B mice. RLIP76(−/−) mice were significantly more sensitive to radiation than the wildtype, and the levels of HNE and GS-HNE were significantly increased in RLIP76(−/−) tissues [226].

Recent studies in the field of chemo- and radioresistance regard the cancer stem cells (CSCs) or cancer-initiating cells, which are a group of cells with self-renewal and differentiation capacity. CSCs are believed to be the cause of chemo- and radioresistance and disease recurrence in many, if not all, types of cancer [227]. Although the redox status of cancer stem cells is not well characterized, it has been suggested that cancer stem cells share low level of ROS [228]. It has been demonstrated that breast cancer stem cells are more tumorigenic and are relatively resistant to radiation at the DNA and cellular levels, due to significantly lower levels of basal and radiation-induced ROS in these cells, indicative of higher levels of ROS scavengers [229]. Human gastrointestinal cancer stem cells with a high level of CD44 expression showed an enhanced capacity of GSH synthesis and defense against ROS by a cysteine-glutamate exchange transporter [230]. Diehn et al. (2009) reported that ROS levels are lower in human and murine breast cancer stem cells compared to nonstem breast cancer cells [231]. In general, breast cancer stem cells display an upregulation of ROS-scavenging molecules that maintain ROS levels to be low, thereby contributing to tumor radioresistance. In a recent report, Cipak et al. demonstrated that HNE and hydroxyl radical-modified collagen cause growth suppression of human breast carcinoma stem cells [232]. In looking specifically at leukemic stem cells (LSCs), several reports suggest the possibility of eradicating LSCs by increasing the ROS level. Guzman et al. (2005) showed that parthenolide induces apoptosis of LSCs in acute myelogenous leukemia (AML) and blast crisis chronic myelogenous leukemia (CML) by increasing ROS levels [233]. Ito et al. (2008) demonstrated an important role of the promyelocytic leukemia protein (PML) tumor suppressor in the maintenance of quiescent CML stem cells, introducing the possibility of eradicating CML stem cells with arsenic trioxide (As_2_O_3_) [234], an ROS generator that inhibits PML. Not only ROS inducers have been considered for eradicating leukemic stem cells, but also HNE has been suggested as a new agent able to effectively eradicate AML at the bulk, progenitor, and stem cell level [106], indicating that the modulation of the redox systems in cancer stem cells is now an active area of research, as it is critical that we begin to develop strategies to eradicate these specific cell populations.

## 10. Concluding Remarks

In recent years, it has become evident that targeting oxidative stress levels and the products of breakdown of polyunsaturated fatty acid is a feasible therapeutic approach that may improve therapeutic selectivity and overcome drug resistance. Numerous agents, that can interfere with redox cell signaling pathways, have been identified, demonstrating, in preclinical models, selective toxicity toward the cancer cells, with increased endogenous ROS, raising oxidative stress over the threshold of toxicity. Association of chemical or radiation therapies with pharmacological agents that have pro-oxidant properties or are able to induce lipid peroxidation increases the effectiveness of the treatment. The increased intracellular antioxidant capacity is a common phenomenon in tumor cells resistant to many anticancer agents and radiation. In these cells, the use of agents to abrogate such adaptation mechanisms in combination with conventional chemotherapy or radiotherapy can improve therapeutic outcomes. Analogous results have been obtained in cancer stem cells, which as well as the chemo- and radioresistant tumor cells have high level of antioxidant capacity. 

The potential of using a redox-modulating strategy to eliminate malignant cells without killing normal cell represents a new therapeutic strategy even if a more detailed understanding of ROS-mediated signaling in tumor cells and in tumor stem cells is necessary to develop the therapeutic intervention to selectively kill cancer cells and overcome drug and radiation resistance.
